# Comparison of Gingival Recession of Mandibular Incisors of Class III Patients Immediately after Compensatory or Surgical Orthodontic Treatment

**DOI:** 10.1055/s-0042-1758068

**Published:** 2022-12-27

**Authors:** Fábio Jorge Saab, Daniel Salvatore de Freitas, Paula Cotrin, Renata Cristina Oliveira, Fabricio Pinelli Valarelli, Ricardo Cesar Gobbi de Oliveira, Samira Salmeron, Célia Regina Maio Pinzan Vercelino, Karina Maria Salvatore Freitas

**Affiliations:** 1 Orthodontic Graduate Student, Ingá University Center UNINGÁ, Maringá, Brazil; 2 Department of Oral Maxillofacial Surgery, Freitas Dentistry Institute, Bauru, Brazil; 3 Department of Orthodontics, Ingá University Center UNINGÁ, Maringá, Brazil; 4 Department of Periodontics, Ingá University Center UNINGÁ, Maringá, Brazil

**Keywords:** Class III malocclusion, gingival recession, corrective orthodontics, orthognathic surgery

## Abstract

**Objective** This study aimed to compare gingival recession in mandibular anterior teeth in patients with Class III malocclusion, immediately after compensatory or surgical orthodontic treatment.

**Materials and Methods** The sample consisted of 40 patients with Class III malocclusion, divided into two groups: Group 1 (compensatory), 20 patients treated with compensatory orthodontics, with a mean initial age of 20.26 years (standard deviation [SD] . = 7.44), mean final age of 23.07 years (SD = 7.32), and mean treatment time of 2.81 years (SD =0.84). Group 2 (surgical), who undergone orthodontic–surgical treatment, with a mean initial age of 23.08 years (SD =5.48), mean final age of 25.43 years (SD =5.12), and mean treatment time of 2.35 years (SD =1.56). Intraoral photographs taken before and after removal of the fixed orthodontic appliance were used to measure the gingival recession, from the cervical of the mandibular incisors from the most cervical point of the gingival margin to the cementoenamel junction. In the initial and final cephalograms, the position of the mandibular incisors was measured. The intergroup comparison was performed using the independent *t* -test.

**Results** The results showed that there was no statistically significant difference in the gingival recession at the beginning, at the end, and of changes with treatment between the compensatory and surgical groups.

**Conclusion** It was concluded that the compensatory and surgical orthodontic treatments for Class III malocclusion showed similar results regarding the gingival recession of the mandibular incisors.

## Introduction

Gingival recession is a common feature of periodontal disease and an undesirable condition. 1 Its definition is the apical displacement of gingival tissues, leading to exposure of the cementoenamel boundary. 2 3 4 5 It can lead to sensitivity and root caries 2 in addition to loss of tooth support, 4 5 which may occur in isolated or generalized areas, affecting at least one tooth surface. 6 Teeth more susceptible to recession gingival are the lower incisors, probably due to the thin, or often nonexistent, bony lamina covering the buccal surface of these roots, in addition to a small, or even absent, band of keratinized gingiva, common in buccal teeth. The buccal surface is more frequently affected than the lingual surface. 3 Gingival recession affects a significant proportion of the population, according to the systematic review by Heasman et al 7 showing an increase in its prevalence as age increases, 2 3 8 9 10 being more prevalent in individuals over 50 years old, 10 without sex differences. 11 12 According to Kassab and Cohen, 2 more than 50% of the population have some degree of gingival recession, which has been found both in individuals with good and poor oral hygiene and more often on the buccal surfaces of the teeth.

The interrelationship between orthodontic movement and gingival recession has been a much-discussed and controversial issue in the orthodontic and periodontal literature. 13 Once established, gingival recession can cause several problems, both aesthetic and functional to the patient. 14 15 Possible damage to periodontal structures caused by orthodontic movement has been the subject of several discussions and research in the areas of orthodontics and periodontics. 2 5 6 8 10 16

The treatment of Class III malocclusion is a challenge for orthodontists, 17 18 mainly, concerning the ideal position of the mandibular incisors, as well as stability and periodontal condition. Patients can be treated basically by two different approaches: the first, correcting only the dentoalveolar disharmony, performing an orthodontic camouflage treatment, with the aid of Class III elastics, with dental compensations, maintaining or exacerbating the naturally lingual position of the mandibular incisors; or performing a treatment with orthognathic surgery combined with conventional orthodontic treatment, decompensating the naturally retro-inclined mandibular incisors, achieving normal occlusion and improved facial aesthetics, 19 20 in addition to an anteroposterior correction of the skeletal bases. In practice, the choice of treatment to be performed depends on several factors, including clinical and radiographic examination, patient complaints, especially in relation to facial aesthetics, financial condition to perform oral and maxillofacial surgery, among others.

The relationship between gingival recession and orthodontic movement is much discussed and controversial. Some studies report that tooth movement beyond the limits of the alveolar bone of the mandibular incisors predisposes to loss of gingival attachment via the buccal, leading to gingival recession. 16 21 22 Others report that there is no evidence linking tooth movement to the development of gingival recession, 11 23 24 but one thing is unanimous: once established, gingival recession can cause several problems, both aesthetic and functional to the patient. It is known to say that orthodontic forces can move roots close to or through the cortical bone, leading to bone dehiscence and Class III skeletal malocclusion, due to the retroclinated positioning of the mandibular incisors in an attempt to compensate for the sagittal discrepancy, is considered a predominant factor for connective tissue loss in the frontal region of the mandible. 9 Dental compensations, with proclinated maxillary incisors and retroclinated mandibular incisors, are common features in patients with Class III malocclusion and help maintain function and mask the skeletal discrepancy. 25

Surgical orthodontic treatment of patients with Class III malocclusion involves orthodontic decompensation of the mandibular incisors, followed by surgical correction of the skeletal discrepancy. Excessive proclination of mandibular incisors performed to decompensate them during presurgical orthodontics can also cause alveolar bone loss around the incisors, bone fenestration, and gingival recession; therefore, special care should be considered in individuals with mandibular prognathism. 26 Compensatory orthodontic camouflage treatment of Class III malocclusion is mainly related to mandibular anterior retrusion and protrusion of maxillary anterior teeth. It is believed that tooth movement toward the tongue can decrease the risk of developing recession, as it affects the migration of the gingival edge toward its crown and causes the growth of the gingiva in height and the alveolar bone. 27

Surgical orthodontic treatment of Class III patients involves orthodontic decompensation of the mandibular incisors, followed by surgical correction of the skeletal discrepancy. Excessive proclination of mandibular incisors performed to decompensate them during presurgical orthodontics can also cause alveolar bone loss around the incisors, bone fenestration, and gingival recession; therefore, special care should be considered in individuals with mandibular prognathism. 26 This movement of the teeth toward the lips is traditionally considered a high-risk parameter for the development of recession. 23

However, few studies on alveolar bone alteration in the mandible, bone dehiscence, and gingival recession in patients with mandibular prognathism undergoing orthognathic surgery or treated with compensatory treatment are available in the literature. 26 28 29 30

Therefore, this study aimed to compare gingival recession in mandibular incisors immediately after compensatory or surgical treatment of Class III malocclusion.

## Materials and Methods

This work was approved by the Research Ethics Committee of the Ingá University Center UNINGÁ, under the number 34185120.5.0000.5220.

The sample size calculation was based on an α significance level of 5% (0.05) and a β of 20% (0.2) to achieve a test power of 80%, and to detect a minimum difference of 0.6mm with a standard deviation of 0.66mm for the gingival recession of the tooth 41. 21 Thus, the sample size calculation indicated the need for 20 individuals in each group.

The inclusion criteria were as follows:

1. Class III molar relationship of any severity at the start of treatment,
2. Complete permanent denture up to the first molars, without supernumeraries or agenesis,
3. Absence of previous orthodontic treatment,
4. Absence of tooth extractions, except for second or third molars,
5. Class III correction planning with fixed orthodontic appliances with prescription Roth or McLaughin, Bennett and Trevisi (MBT) brackets,
6. Complete initial and final orthodontic records and in good condition for evaluation,
7. Patients with healthy periodontium, without signs of hypertrophied gingiva in the region of the lower incisors in the initial and final photographs,
8. Patients with no history of systemic diseases or who used medications that could change the gingival condition,
9. Well-finished cases, with Class I canine and molar relationships,
10. Cases treated without interproximal stripping.

The sample consisted of the documentation of 40 patients with Class III malocclusion treated with fixed orthodontic appliances associated or not with orthognathic surgery selected from the archives of from the archives of IOPG and IOFreitas, Bauru, SP, Brazil.

The records contained initial and final intraoral photographs of the orthodontic treatment, and the final photographs were taken at least 1 month after the removal of the orthodontic appliance, initial and final cephalograms, and initial dental or digital models. Data regarding age, sex, and duration of treatment were obtained from the patients' medical records.

Patients were divided into two groups, according to the type of treatment performed:

- Group 1 (compensatory): 20 patients treated with fixed appliances, biofunctional prescription, Roth and MBT, and Class III intermaxillary elastics, 12 females and 8 males, with a mean initial age of 20.26 years (standard deviation [SD] = 7.44), mean final age of 23.07 years (SD =7.32), and mean treatment time of 2.81 years (SD =0.84). The mean initial mandibular anterior crowding was 1.23 mm (SD = 0.90). Alignment was performed with nickel–titanium round archwires, leveling with stainless steel archwires and orthodontic mechanics with 0.019”x0.025” rectangular stainless steel archwires with the aid of Class III intermaxillary elastics.
- Group 2 (surgical): 20 patients treated with fixed appliances, Roth or MBT prescription, associated with orthognathic surgery, 9 females and 11 males, with a mean initial age of 23.08 years (SD = 5.48), mean final age of 25.43 years (SD = 5.12), and mean treatment time of 2.35 years (SD = 1.56). The mean initial mandibular anterior crowding was 1.38 mm (SD = 1.45). Alignment was performed with nickel–titanium round archwires, leveling with stainless steel archwires and after installation of 0.019”x0.025” rectangular stainless steel archwires, the patients underwent orthognathic surgery. The buccal tipping and decompensation of the mandibular incisors in the presurgical phase occurred either with the aid of Class II elastics or by performing buccal torque on the 0.019x.025 rectangular archwires. After the orthognathic surgery was performed, retention mechanics with intermaxillary intercuspation elastics and removal of the orthodontic appliance were performed.

All patients from both groups received, after treatment, oral hygiene care instructions, such as chairside verbal instructions about toothbrushing at least three times per day, daily ﬂossing, and chlorhexidine mouth rinsing when necessary.

### Analysis of Photographs

The classification of the degree of malocclusion severity, determined by the molar relationship, in the initial and final stages of the treatment, was estimated in the lateral intraoral photographs.

The molar relationship of each patient was classified as ¼-cusp, half-cusp, ¾-cusp, or complete Class III, bilaterally. Each patient was given a score for the molar relationship, adding the classification of both sides and dividing by 2. In cases of intermediate results, the score/value was rounded up.

**Fig. 1:**
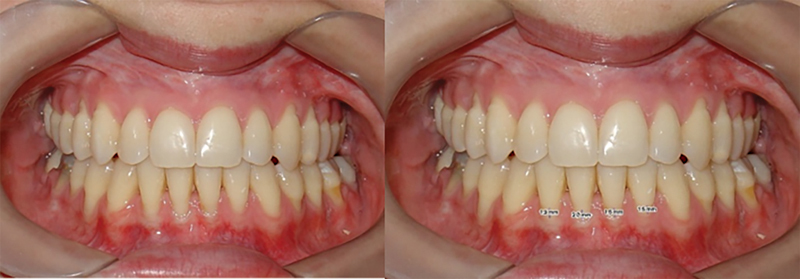
Measurement of the gingival recession of the four mandibular anterior teeth.

Gingival recession was measured on frontal intraoral photographs on the buccal surface of the four mandibular incisors. 31 In the first stage, each mandibular incisor was analyzed for the presence or absence of gingival recession. When the cementoenamel junction was not exposed, a score of 0 was assigned to that tooth. The others that showed some degree of recession were reassessed and, for each tooth, a linear measurement was made from the most cervical point of the gingival margin to the cementoenamel junction ( Fig. 1 ). Patients in which the cementoenamel junction was not visible in the intraoral frontal photographs were excluded. The measurement was performed using the Dolphin program (version 11.95, Dolphin Imaging & Management Solutions, Chatsworth, California, United States), the dots per inch (DPIs) of each photograph as calibration, performed by the software. 31

Measurement of gingival biotype was also performed on initial frontal intraoral photographs of each patient, and classified as thin-scalloped, thick-scalloped, and thick-flat gingival biotype. 32 33

### Cephalometric Analysis

The evaluation of the initial and final cephalometric characteristics of each patient was determined by the lateral cephalometric radiographs obtained at the beginning and the end of orthodontic treatment. The initial and final cephalograms of each patient were scanned, digitized, and entered into the Dolphin Imaging Premium 11.95 program (Dolphin Imaging & Management Solutions, Chatsworth, California, United States).

After measurements were performed, the following cephalometric variables were compared:

- 1-NB (mm): Distance from the most buccal portion of the lower incisor crown to the NB line. It indicates mandibular incisor protrusion.
- 1.NB (degrees): Angle between the long axis of the lower incisor and the NB line. It relates the inclination of this tooth with the mandible and the nasion;
- IMPA (degrees): Angle between the long axis of the lower central incisor and the GoMe mandibular plane. It indicates the inclination of this tooth in relation to the mandible.

### Model Analysis

**Fig. 2:**
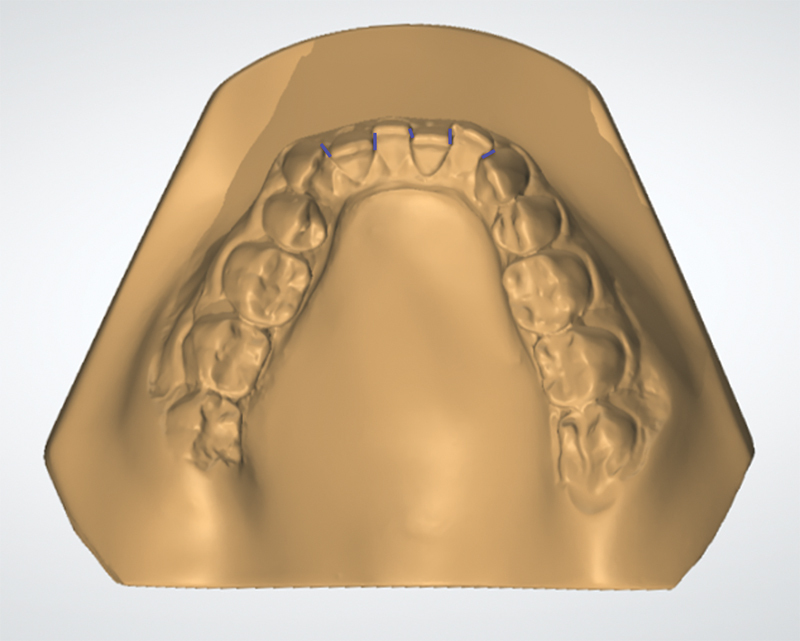
Measurement of Little's irregularity index.

On the initial dental cast of each patient, the Little's irregularity index 34 was measured, which is the sum of the distance between the contact points of the six mandibular anterior teeth, indicating the amount of initial crowding ( Fig. 2 ). This measurement was performed by a single examiner previously calibrated with the aid of a digital caliper (Hangzhou Hantoo Enterprises, Hangzhou, China), positioned parallel to the ground.

### Error Study

To calculate the method error, the lateral cephalograms, gingival recession, and Little's irregularity index of 8 patients were remeasured with a time interval of 30 days. The casual error was determined using the Dahlberg formula and the systematic error using the dependent *t* -test, with a significance level of 5% ( *p* < 0.05). The gingival biotype was also reassessed after 1 month in eight patients, and the error was calculated using the Kappa test.

### Statistical Analysis

The Shapiro–Wilk test was used to assess data normality. To verify the compatibility of the groups in terms of sex distribution, Class III malocclusion severity, and gingival biotype, the chi-squared test was performed. To compare age, treatment time, and Little's irregularity index, the independent *t* -test was used.

Intergroup comparison of initial and final gingival recession and changes with treatment was performed using the independent *t* -test. The tests were performed using the Statistica software for Windows (version 12.0, Statsoft, Tulsa, Oklahoma, United States), considering the significant results for *p* less than 0.05.

## Results

There was no significant systematic error and random errors ranged from 0.00 (tooth 31) to 0.32 mm (tooth 32) and from 0.33 (1.NB) to 0.54 degrees (IMPA). The results of the Kappa test in relation to the gingival biotype showed a coefficient of 0.89, indicating an almost perfect correlation.

Initial and final ages, treatment time, mandibular anterior crowding, gender distribution, Class III severity, and gingival biotypes were compatible between the two groups ( Table 1 ).

**Table 1:** Comparability of groups of initial and final ages, treatment time, and sex distribution (independent *t* -test and chi-square test)

Variables	Compensatory *n* = 20		Surgical *n* = 20		*p* -Value
	Mean	SD	Mean	SD	
Initial age (years)	20.26	7.44	23.08	5.48	0.182
Final age (years)	23.07	7.32	25.43	5.12	0.246
Treatment time (years)	2.81	0.84	2.35	1.56	0.378
Little's irregularity index (mm)	1.23	0.90	1.38	1.45	0.696
Sex					
Males	8		11		X 2 = 0.90 DF = 1 *p* = 0.342
Females	12		9		
Class III severity					
¼-cusp	4		4		X 2 = 0.52 DF = 3 *p* = 0.915
Half-cusp	4		3		
¾-cusp	6		7		
Full-cusp	6		6		
Gingival biotype Thin-scalloped	4		4		X 2 = 0.82 DF = 2 *p* = 0.664
Thick-scalloped	12		14		
Thick-flat	4		2		

Abbreviation: SD, standard deviation.

When comparing the gingival recession and the position of the incisors in the initial and final phases between the two groups, there was no statistically significant difference ( Table 2 ). There was no difference between the groups in the change in the amount of gingival recession with treatment ( Table 2 ). The surgical group showed significantly greater proclination of the mandibular incisors with treatment than the compensatory group, which showed slight retroclination with treatment ( Table 2 ).

**Table 2:** Intergroup comparison of gingival recession at the initial (T1) and final stage (T2) and treatment changes (T2-T1) (independent *t* -test)

Variables	Compensatory *n* = 20		Surgical *n* = 20		*p* -Value
	Mean	SD	Mean	SD	
**Initial (T1)**					
**Gingival recession**					
Tooth 32 (mm)	0.24	0.26	0.28	0.36	0.690
Tooth 31 (mm)	0.23	0.20	0.45	0.59	0.123
Tooth 41 (mm)	0.24	0.25	0.37	0.45	0.264
Tooth 42 (mm)	0.19	0.19	0.32	0.28	0.091
**Cephalometric variables**					
1-NB (mm)	4.48	2.51	4.13	4.34	0.756
1.NB (degrees)	25.41	5.58	25.19	4.48	0.891
IMPA (degrees)	88.06	8.42	83.03	7.45	0.053
Final (T2)					
**Gingival recession**					
Tooth 32 (mm)	0.42	0.35	0.35	0.48	0.625
Tooth 31 (mm)	0.38	0.35	0.59	0.69	0.233
Tooth 41 (mm)	0.33	0.28	0.42	0.45	0.478
Tooth 42 (mm)	0.27	0.31	0.38	0.45	0.375
**Cephalometric variables**					
1-NB (mm)	4.48	1.89	4.08	2.04	0.524
1.NB (degrees)	24.47	4.32	24.03	5.40	0.778
IMPA (degrees)	87.52	6.27	87.48	7.19	0.987
Treatment changes (T2-T1)					
**Gingival recession**					
Tooth 32 (mm)	0.18	0.20	0.08	0.38	0.281
Tooth 31 (mm)	0.15	0.29	0.14	0.35	0.922
Tooth 41 (mm)	0.09	0.30	0.05	0.22	0.592
Tooth 42 (mm)	0.08	0.26	0.06	0.28	0.816
**Cephalometric variables**					
1-NB (mm)	0.00	1.73	−0.05	3.83	0.958
1.NB (degrees)	−0.94	6.21	−1.16	6.44	0.913
IMPA (degrees)	−0.54	6.45	4.46	5.64	** 0.013 a**

Abbreviations: IMPA, incisor mandibular plane angle; NB, line Nasion to point B; SD, standard deviation.

a Statistically significant for *p* < 0.05.

## Discussion

The selection of the sample was performed to compare which of the protocols of Class III treatment, whether camouflage or orthosurgical, cause less gingival recession so that the treated groups were as compatible as possible.

The criterion chosen to assess the anteroposterior severity of malocclusion was the molar relationship, unlike some studies that chose to assess cephalometric characteristics to determine the sagittal discrepancy of patients. 28

The absence of tooth extractions, as well as the presence of all permanent teeth up to the first molars and no interproximal stripping performed during orthodontic treatment, were also requirements in the inclusion criteria of the samples so that cases treated with retraction of the anterior battery were naturally excluded since this mechanics has a direct consequence on the periodontium teeth support. 35

Another basic requirement for inclusion in the sample was the absence of periodontal disease, an item that proved to be common in many similar studies, 21 23 24 since the active periodontal disease has a direct influence on the gingival condition. 8 21

It is a consensus in the literature that gingival recession is directly linked to the age of the individual, and its prevalence and severity are related to aging, 2 3 8 9 10 with no predilection for sex. 11 12 In this study, the groups were compatible in terms of initial and final ages and sex distribution.

The presence of the fixed appliance causes greater accumulation and retention of plaque, in addition to increasing the difficulty of cleaning 36 hence, the concern with compatibility between the two groups in this study, with no statistically significant difference between them.

The experimental groups showed compatibility and did not present statistically significant differences; however, the concern with the initial severity of the malocclusion was because the greater the Class III relationship, the greater the tooth movement and it is inanimate to say that tooth movement beyond the alveolar bone boundaries of the mandibular incisors predisposes to loss of gingival attachment, leading to gingival recession. 16 21 22

Knowing that the teeth most susceptible to gingival recession are the mandibular incisors and that the buccal surface is more frequently affected than the lingual surface, 3 the measurement in absolute values was performed directly on the frontal intraoral photographs; these values were compatible between the groups.

The gingival biotypes of the samples were compared through frontal intraoral photographs and classified into thin-scalloped, thick-scalloped, and thick-flat gingival biotypes, 32 33 with compatibility between the compensatory and surgical groups. It is of fundamental importance to know how to identify the gingival biotype, as it is known to say that thinner gingiva is more susceptible to gingival recession, while thicker gingiva is less susceptible. 33

The compatibility between the groups is of fundamental importance in conducting research, and gingival recession, the main object of study of this research, was compatible in the initial phase (T1) in both groups, compatibility also present when evaluated in an intergroup comparison of changes in gingival recession with orthodontic treatment (T2-T1), confirming that there is no statistically significant difference when evaluating gingival recession in patients treated in compensatory or orthosurgical protocols. Studies have evaluated gingival recessions in Class III patients treated compensatorily 25 and surgically, 16 26 30 but little or nothing is known about a comparison between these two protocols of treatment.

The ideal would be to measure the gingival recession directly in the mouth, but as this study was retrospective and this measurement was not included in the patients' medical records, the only possible means of analysis would be to exclude all patients with periodontal disease, initial and severe initial recession degree 21 and call patients for final recession measurement. However, it would only be possible to call patients who had recently completed the treatment, as aging directly influences the increase in gingival recession. 8 9 23 All these factors would greatly reduce the sample, possibly making this study unfeasible. This way, gingival recession was evaluated immediately after orthodontic compensatory or surgical treatment, due to the retrospective design of the study.

The amount of gingival recession was measured in frontal intraoral photographs on the buccal aspect of the four mandibular incisors. In a first moment, each mandibular incisor was analyzed to verify the presence or absence of gingival recession and in a second moment, the incisors that presented some degree of gingival recession were reassessed and, for each tooth, the measurement of the most cervical point was made from the gingival margin to the cementoenamel junction. The measurement was performed using the Dolphin program and the calibration was performed using the DPIs of each photograph. 31

Final photographs used were obtained at least 1 month after appliance removal, as final photographs were taken immediately after appliance removal could show signs of inflammation and gingival swelling characteristic in patients who wear orthodontic appliances, especially younger ones. 37

Due to the change in the position of the mandibular incisors generated in orthodontic treatment that could be related to gingival recession, most studies that evaluate periodontal changes in patients undergoing orthodontic treatment also perform cephalometric measurements for this comparison, as performed in this work. 21 23 24 38

The degree of mandibular anterior crowding was evaluated in the initial dental models by the Little's irregularity index 34 this index is widely used and easily reproducible. 21 31

The intergroup comparison of gingival recession between the compensatory and surgical groups in the initial and final treatment changes did not show statistically significant differences ( Table 2 ). Some previous studies in Class III patients treated surgically and/or compensatory also showed that there was no significant change in the gingival margin. 17 26 39

Patients in the surgical group had, on average, significantly greater proclination of the mandibular incisors with treatment than the compensatory group, which showed slight retroclination with treatment ( Table 2 ). This can be explained by the side effect of the use of Class II elastics during orthodontic preparation for orthognathic surgery, promoting proclination of the mandibular incisors. 16 40 Some studies found an association between alveolar bone loss around the mandibular incisors after excessive buccal movement. 21 26 On the other hand, the compensatory group showed minimal and nonsignificant retroclination of the mandibular incisors with treatment ( Table 2 ), probably due to the use of Class III elastics to correct this malocclusion in a nonsurgical way. 17 18 41

Choi et al 30 evaluated Class III patients undergoing presurgical treatment comparing cases that suffered decompensation with great proclination of the mandibular incisors and cases that were decompensated but with minimal change in the inclination of the mandibular incisors. In both groups, clinical crown length and probing depth increased during preoperative treatment. The width of the attached gingiva decreased more in the group with a higher slope of the mandibular incisors than in the group with less inclination. They then concluded that greater buccal tipping of the mandibular incisors in presurgical treatments in Class III patients results in decreased buccal alveolar bone and a consequent decrease in attached gingiva, however, without clinical significance. 30 However, in this study, there was no greater increase in gingival recession in surgical cases, which suffered incisor decompensation before orthognathic surgery. Corroborating the results of this study, some studies found no association between the buccal movement of mandibular incisors induced by the orthodontic appliance and gingival recession. 38 42 According to Aziz and Flores-Mir, 42 factors that can lead to gingival recession after orthodontic tilting and/or translational movement are the reduced thickness of the free gingival margin, a narrow mandibular symphysis, poor plaque control, and aggressive tooth brushing. 42

Regardless of the treatment used to correct Class III malocclusion, care should be taken with the patient's initial periodontal situation, and avoid excessive buccal movement of the mandibular incisors, which seems to increase the chance of gingival recession. 30 31 In cases of patients who present gingival recession before starting treatment or with other periodontal problems, bone loss, or other limitations, greater care should be taken, as this periodontal condition may worsen. 9 43 44

## Conclusion

Patients with Class III malocclusion treated compensatory or surgically show a similar change in mandibular incisor gingival recession immediately after treatment.
